# A pilot study of Aboriginal health promotion from an ecological perspective

**DOI:** 10.1186/1471-2458-11-749

**Published:** 2011-09-30

**Authors:** Rachel E Reilly, Marion Cincotta, Joyce Doyle, Bradley R Firebrace, Margaret Cargo, Gemma van den Tol, Denise Morgan-Bulled, Kevin G Rowley

**Affiliations:** 1 Onemda VicHealth Koori Health Unit, Centre for Health and Society, School of Population Health, the University of Melbourne VIC 3010, Australia; 2 Victorian Aboriginal Community Controlled Health Organisation, Fitzroy VIC 3065, Australia; 3 Rumbalara Aboriginal Co-operative, Mooroopna VIC 3629, Australia; 4 Rumbalara Football Netball Club, Shepparton VIC 3630, Australia; 5 School of Health Sciences, University of South Australia, Adelaide SA 5001, Australia; 6 Viney Morgan Aboriginal Medical Service, via Barmah VIC 3639, Australia

## Abstract

**Background:**

For health promotion to be effective in Aboriginal and Torres Strait Islander Communities, interventions (and their evaluation) need to work within a complex social environment and respect Indigenous knowledge, culture and social systems. At present, there is a lack of culturally appropriate evaluation methods available to practitioners that are capable of capturing this complexity. As an initial response to this problem, we used two non-invasive methods to evaluate a community-directed health promotion program, which aimed to improve nutrition and physical activity for members of the Aboriginal community of the Goulburn-Murray region of northern Victoria, Australia. The study addressed two main questions. First, for members of an Aboriginal sporting club, what changes were made to the nutrition environment in which they meet and how is this related to national guidelines for minimising the risk of chronic disease? Second, to what degree was the overall health promotion program aligned with an ecological model of health promotion that addresses physical, social and policy environments as well as individual knowledge and behaviour?

**Methods:**

Rather than monitoring individual outcomes, evaluation methods reported on here assessed change in the nutrition environment (sports club food supply) as a facilitator of dietary change and the 'ecological' nature of the overall program (that is, its complexity with respect to numbers of targets, settings and strategies).

**Results:**

There were favourable changes towards the provision of a food supply consistent with Australian guidelines at the sports club. The ecological analysis indicated that the design and implementation of the program were consistent with an ecological model of health promotion.

**Conclusions:**

The evaluation was useful for assessing the impact of the program on the nutrition environment and for understanding the ecological nature of program activities.

## Background

In the Goulburn-Murray region of northern Victoria, Aboriginal People are at increased risk of serious health problems as a result of complex social and historical processes [1,2]. This population suffers a burden of ill-health and socio-economic disadvantage following a similar pattern to other less well-resourced regions of Australia [3]. In particular, rates of preventable disease - diabetes, metabolic syndrome and cardiovascular disease - are high [3,4]. Dietary quality is widely recognised as a contributor to Aboriginal ill-health, including in this location [5,6], and is determined by individual knowledge, social norms and available food supply. The need for community owned and directed, culturally appropriate interventions that promote nutrition and physical exercise to minimise risk of chronic disease has been identified within this community [5]. Culturally appropriate interventions take account of Aboriginal models of health and social determinants, and the wider social context of the everyday lives of Aboriginal people within which 'health behaviours' take place [7].

Within the expanding discourse on determinants of health there is an acknowledgement that attributing health problems of Aboriginal groups to social disadvantage alone does not account sufficiently for the high rates of preventable disease in Aboriginal population groups [8-10]. There are unique historical, cultural and social determinants, including but not limited to the marginalised position of Aboriginal people in relation to mainstream Australian society, that feature prominently in literature as contributors to the health of Aboriginal and Torres Strait Islander peoples and communities [1,2,10,11]. In addition, for Aboriginal people the definition of health is broad, including the social, spiritual, emotional and physical wellbeing of the whole community [12].

Aboriginal-led health promotion programs acknowledge and work to this definition, explicitly or otherwise, but there is a gap in knowledge on how to evaluate the extent to which activities have addressed it. Accordingly, practitioners have identified a need to develop methods that come closer to measuring the impact of interventions, which are often complex and multifaceted [13]. We argue that an ecological approach has the potential to meet this need as it is more aligned with the holistic, 'whole of community' approach favoured by Aboriginal Community Controlled Organisations in the target community.

Ecological theory builds on the psychosocial determinants framework by providing a systematic way of describing communities as multi-level, with interactions between the social, physical and policy systems in which people live [14,15]. Like the psychosocial determinants framework, ecological theory recognises that the influence of the social and physical environment is crucial to wellbeing. Since the 1990s the adoption of ecological frameworks for targeting and evaluating health promotion interventions has gained momentum as methods for planning and assessing ecological interventions have been developed [16-20].

Community practitioners have advocated for health programs that address the causes and correlates of Aboriginal wellbeing, particularly those identified locally [1,2]. They also seek to evaluate programs in terms of how these determinants are impacted upon. Ecologically-based intervention and evaluation more easily allow analysis of health outcomes in the context of their social determinants, and can identify effective points of intervention in social determinants. Methods developed for this purpose [eg. [21]] are non-invasive, accessible and easily adopted by health promotion practitioners. These are important considerations in the local Aboriginal community context where there is a preference away from the collection of personal individualised information for research purposes and where capacity for evaluation has been low [22].

## Aims

The work described here was a collaboration between three Aboriginal community-controlled organisations and a University, as part of a state-government funded program promoting nutrition and physical activity. The program was funded to: a) evaluate the dietary Guidelines for Australian Adults [23] and the National Physical Activity guidelines for Australians [24] with respect to their rationale, clarity, means of communication and feasibility of implementation for Aboriginal and Torres Strait Islander people in the Goulburn-Murray region; b) develop alternative means of communicating key messages from the national guidelines that are meaningful to and can be acted upon by Aboriginal and Torres Strait Islander people, given the prevailing economic, social and cultural environment; and c) evaluate these novel health promotion tools with respect to the accuracy of the messages received from them by Aboriginal and Torres strait Islander people and their effect on health behaviours. In practice, aims b) and c) were modified through participatory research processes [25] to address issues of diet and exercise in a manner that was more aligned with the principles, practices and current priorities of the participating Aboriginal organisations. Rather than developing messages based on the guidelines, the community organisations sought to implement and evaluate interventions that responded more directly to community needs. This process is discussed in detail in an earlier publication [22]. Hence this prospective study aimed to pilot the use of two non-invasive, ecological or 'system-level' measures to evaluate a series of health promotion activities implemented in a northern Victorian Aboriginal community.

The specific questions addressed in the present study were: 1) for members of the sporting club, what changes were made to the club environment with respect to nutrition and dietary quality and how do they relate to national guidelines for minimising the risk of chronic disease?; and 2) To what degree was the overall health promotion program aligned with an ecological model of health promotion, which addresses the physical, social and policy environments as well as individual knowledge and behaviour?

## Methods

### Setting

The semi-urban Aboriginal population of this region is spread across and between three regional centres close to the convergence of the Goulburn and Murray Rivers in northern Victoria. These are Shepparton, a regional city, Mooroopna, a smaller town, and Cummeragunja, an Aboriginal township. The Aboriginal population is estimated to be greater than 1820, making it the largest non-metropolitan Aboriginal population in Victoria [26]. This research took place in three key community organisations: the Rumbalara Football Netball Club (RFNC), an Aboriginal sporting club accessed by community members of all ages for sport, social events and other health-related activities; the Rumbalara Aboriginal Co-operative, a large provider of medical and social services; and the Viney Morgan Aboriginal Medical Service, located at Cummeragunja, approximately 65 km north of the regional centre, Shepparton. In addition to providing essential services, these Community-Controlled Organisations, which were established on the basis of Aboriginal resistance to discriminatory policies and practices, are centres of community activity, connection, belonging and identity for the Goulburn-Murray Aboriginal community [1]. Available data indicate that the majority of community members access one or more of these partner organisations [27,28].

### Program development and activities

The health promotion program was developed in response to community needs identified in prior research [5,22]. Activities were implemented by local health workers employed within the partner organisations, together with university researchers. All activities developed as part of the State-funded project were included in the analysis. Discrete activities were implemented with Elders (n = 15), women (n = 25 per week), youth (junior footballers, n = 40), employees at the Rumbalara Co-operative (n = 20) and all those accessing the Rumbalara Football Netball Club for training, other programs and game days (n = approximately 600 per week). The activities did not specifically target those at risk of or suffering disease.

Specific details of program activities have been described elsewhere [22,29]. Briefly, program activities were: a health 'summer school' for health promotion practitioners; a nutrition program for under-seventeen year-old footballers; initiatives aimed at improving the dietary quality of food supplied at RFNC; a series of focus groups aimed at adapting mainstream nutrition guidelines for the Indigenous community; a weekly self-directed health-focused meeting for women; and a workplace exercise program. Activities were facilitated by local Aboriginal (in the majority of cases) and non-Aboriginal health promotion practitioners employed by the community organisations.

The work was approved by the University of Melbourne Human Research Ethics Committee and was overseen by a Steering Committee which represented the three Aboriginal partner organisations, the University of Melbourne and the Department of Human Services. University researchers were involved in the process of program development and evaluation as partners within a participatory framework [25]. A Memorandum of Understanding acknowledged control of data and reporting by the Aboriginal organisations.

### Store turnover

The store turnover analysis concerns the Rumbalara Football Netball Club, a significant community gathering place where community members meet regularly for sports training, game-days and other community events. Store turnover monitors nutrient density in the food supply at a specific site and has been shown to accurately reflect biological markers of nutrition at the community level [30]. Changes in the food supply/nutrition environment were measured at RFNC over the course of the winter sport seasons (April - September) of 2005 (pre-intervention) and 2006. This incorporated meals provided to players on match days and at training, a breakfast program for junior players, food sold at the canteen and a 'fruit-share' program. The types and amounts of food and drink purchased for all club activities were derived from the canteen's receipts. Store turnover is a non-invasive measure of the overall nutrition environment that is not subject to the recall biases of individual food intake studies. The nutrient and energy content of food and drink were analysed using FoodWorks [31] and Microdiet [32] programs.

### Ecological analysis

**Figure 1:**
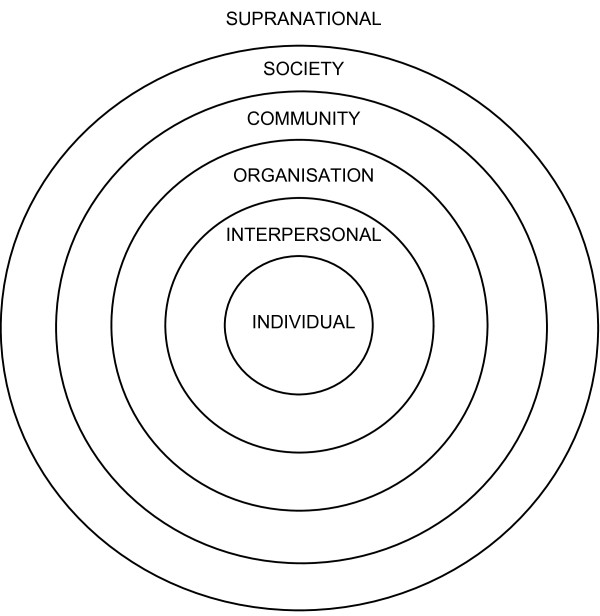
**The six levels of Miller's Systems theory adapted for ecological analysis**. Miller's levels extended to 'organ' and 'cell' [34]. Diagram adapted from [33].

Richard and colleagues [33] provide a procedure to assess the degree to which a health promotion program is 'ecological' or could be improved through better targeting of the social environment. Interventions are coded on a grid according to two dimensions - the *setting *and the *intervention target*. The four higher categories of Miller's Systems Theory [[34]; see Figure 1] are adopted as settings for ecological analysis: organisations, communities, societies and 'supranational systems' (two or more countries). A health promotion intervention may take place in one or more of these settings. Within each setting, a number of targets are possible. For the analysis, five possible targets are defined as the individual (IND), the interpersonal environment (INT), the organisation (ORG), the community (COM) or political targets (POL). To be deemed ecological, an intervention will target the individual and at least one environmental target.

The intervention *strategy *is defined according to the relationships between the intervention and its target(s). The simplest strategy is where the aim of an intervention is to directly change a given target. For example, where intrapersonal health determinants, such as a person's attitudes, knowledge or beliefs, are targeted (HP → IND), or where changes are made to the structure or function of an organisation (HP → ORG). The second level of complexity is where the intervention acts to create networks among two or more targets, for example, a self-help group relying on interaction between clients (HP → [IND-IND]). Other, higher-level intervention strategies might involve lobbying elected officials to make changes to regulations affecting organisations in the client's environment (HP → POL → ORG → IND). There are numerous possible combinations [see [16,33]]. A program is more ecological the more targets it has across a variety of settings.

The overall program comprised six health promotion activities. At the conclusion of each activity, each facilitator was interviewed and completed an activity implementation questionnaire comprising open and close-ended questions based on previous and concurrent studies applying the ecological approach in Canada [16,33] and a remote Aboriginal community in Australia [35]. The questionnaire included an open-ended description of the activity, its objectives, intended beneficiaries (targets) and the settings in which it took place. Data were entered into a spreadsheet, which allowed comparison across different program activities and the collation of summary data relating to the whole program. The two principal academic investigators reviewed each questionnaire and interview report and reached consensus on the coding of each activity. The analysis was then reviewed and approved by program facilitators. Using the framework outlined, the overall health promotion program was assessed to ascertain its ecological 'score' (Table 1).

**Table 1:** Ecological analysis scoring framework

Score	Activity characteristics
0	Only one intervention strategy, independent of number of settings;
1	At least two different intervention strategies, which did not include the direct targeting of the participant, again regardless of the number of settings;
2	One setting in which at least two strategies were implemented, one of which directly targeted the participants;
3	Two settings in which at least two strategies were implemented, one of which directly targeted the participants;
4	Three or more settings in which at least two strategies were implemented, one of which directly targeted the participants.

## Results

### Store turnover: Trends in dietary quality at RFNC canteen

In 2005, the canteen bought apples, pears, oranges, pineapples rockmelons and lettuce. In 2006, these lines were continued, with the addition of mandarins, grapes and honeydew melons, carrots, cauliflowers, tomatoes and onions. There was also a shift to buying 100% fruit juices, which have less sugar and more vitamins than lemonade and cola drinks. Fresh beef and chicken were introduced in 2006. These were barbequed, and salad rolls and sandwiches were made on site.

The food groups supplied for 2005 and 2006 are shown in Table 2. To adjust for variations in total amount sold, we expressed the amount of nutrient or food as a density per megajoule (Mj) offered in that year. For context, observed nutrient densities are reported beside "suggested dietary targets" for lowering risk of chronic disease [36]. In general, the latter equate to the 90^th ^percentile of daily intake in the Australian population, and are thus somewhat higher than conventional 'recommended daily intakes' which relate to prevention of overt symptoms of nutrient deficiency. Observed food group densities are reported alongside NHMRC core food group recommendations [23]. For both food and nutrient variables, a density per Mj consistent with a healthy food supply was calculated by dividing the suggested or recommended value by 10 Mj (that is, an estimate of typical daily energy requirement) [36].

**Table 2:** Trends in food groups and selected nutrients at RFNC canteen.

Food/nutrient	density in food supply, per Mj		density, per Mj reference values^a^
	2005	2006	
*Foods*			
fresh fruit, g	47	80	33
fresh vegetables, g	1.1	2.0	31
breads, flour, g	9.3	17.6	20
fresh meat and eggs, g	1.8	8.4	10
milk and cheese, g	20	24	24-72
pies, pasties, sausage rolls, g	14	10	*n/a*
cakes, sugar, confectionary, g	13	10	*n/a*
*Nutrients*			
fibre, g	2.0	2.5	3.3
vitamin E, μg	0.41	0.25	1.7
vitamin A, μg ^b^	39	32	136
vitamin C, mg	19	31	21
folate, μg	17	25	60
potassium, mg	265	293	470
sodium, mg	364	359	<160

^a ^calculated on the basis of a person consuming 10 Mj of energy per day [36]. For foods, reference values are derived from recommended core food group intakes [23]; for nutrients, reference values are derived from suggested dietary targets for minimal risk of chronic disease [36] (see *Methods*); ^b^as retinol equivalents.

There were increases in the contribution of fresh meat and eggs, fruit, vegetables and bread/flour, and decreases in cakes and confectionary and pies. Micronutrient data showed increased densities of vitamins A and C and folate over time and a small but favourable trend in the ratio of sodium to potassium in the food supply (Table 2). The contribution of macronutrients to energy in 2005 and 2006 are shown in Table 3, next to suggested densities. The change from 2005 to 2006 was towards the recommended macronutrient composition, including a reduction in total fat content to within the recommended range [36] and a fall in total sugar content.

**Table 3:** Contribution to Energy from macronutrients

Macronutrient	contribution to total energy suggested		Suggested target*
	2005	2006	
protein	9.7%	13.5%	15-25%
total fat	37.3%	31.9%	20-35%
saturated fat	15.8%	13.6%	<10%
carbohydrate	53.0%	54.7%	45-65%
sugars	31.4%	23.3%	n/a

*[36].

By 2006, the nutrition environment was consistent with the characteristics of a healthy food supply with respect to fruit, dairy products and total fat and carbohydrate content, and approached recommended contents of bread/flour, meat and protein. Saturated fat content remained higher and fibre lower than recommended. For micronutrients in relation to minimal risk of chronic disease, vitamin C content met suggested targets, while the fat soluble vitamins E and A were lower than optimal in 2005 and decreased in parallel with the fall in total fat. Sodium and potassium remained less than ideal for chronic disease prevention (although potassium content approached the more conservative "recommended daily intake" equivalent of 330 mg/Mj).

### Ecological analysis

Results of the ecological analysis are summarised in Table 4. The coding of each activity is described below. In each case HP refers to the health promotion program and the ultimate target is 'IND' referring to all individuals who are part of the target population: the Goulburn Murray Aboriginal community.

**Table 4:** Characteristics of the six program areas with respect to the ecological model of health promotion

Activity	Settings	Targets	Strategies
*Health Summer School*	Indigenous community (COM)	practitioners themselves	HP → IND
		Organisational capacity	HP → ORG → IND
*Hungry for Victory*	RFNC (ORG)		
Program launch		U17 footballers	HP → IND; HP → [IND-IND]
Breakfast program		U17 footballers	HP → IND; HP → [ORG-ORG] → IND
Nutrition workshops		U17 footballers & netballers	HP → IND
Mentoring program		U17 & U14 footballers	HP → INT → IND
*Fruit Share*	RFNC (ORG)	RFNC attendees, club	HP → IND; HP → ORG → IND
*Focus groups on guidelines*	Indigenous community (COM)	Participants	HP → IND
		Organisational partnership	HP → [ORG-ORG] → IND
*Women's Wellbeing Group*	VMAMS (ORG)	community women	HP → IND; HP → [IND-IND]
		Organisational partnership	HP → [ORG-ORG] → IND
*10-Week body Challenge*	RAC (ORG)	workplace	HP → ORG → IND
		RAC-RFNC outreach	HP → [ORG-ORG] → IND
		RAC staff (IND)	HP → IND
**Total**	**2**		**5**

Health Summer School: a five-day course which provided participants with updated knowledge in nutrition and a forum to develop programs to be implemented back in the community. Since the participants in the summer school were members of the Aboriginal community, and the course acted to increase their nutritional knowledge, this direct effect is coded as HP → IND. The course also acted to increase health promotion program development capacity within the organisations by educating the practitioners to implement programs back in the community. This strategy is coded as HP → ORG → IND.

Hungry for Victory: a nutrition program for junior footballers at RFNC included education, match-day breakfasts and mentoring. It aimed to improve the nutritional knowledge and dietary intake of participants by focusing on the relationship between nutrition and sporting performance. The activity comprised four distinct parts. The first part was a program launch where following speeches each participant was presented with a t-shirt and drink bottle bearing the program logo. This aimed to motivate participants (HP → IND) and foster team spirit (HP → [IND-IND]). The second part involved the provision of a healthy breakfast to the U/14 footballers on home-game days. The objective was both to provide a healthy breakfast (HP → IND) and build friendly relationships between opposition teams (HP → [ORG - ORG] → IND) with the view that this contributes to an environment of healthy eating, which transcends inter-club rivalry. Nutrition workshops were included in the coaching program and aimed to improve knowledge of how nutrition impacts on football performance (HP → IND). The mentoring activity targeted the individual via an interpersonal relationship (HP → INT → IND). In total there were four different strategies used in this activity in one setting (the sporting club);

Fruit-Share aimed to improving the dietary quality of food supplied at RFNC. Fruit was purchased and provided to players and other members on practice and game days (HP → IND), and the nutritional value of food purchased for the canteen was improved. The intermediary target in this case was the organisation (HP → ORG → IND). The activity used two strategies in a single setting.

Focus groups aimed at reviewing and re-designing nutrition guidelines for the target community were conducted with a cross-section of community members in a range of community centres (27 participants, 11 men and 16 women). An educational component to the focus groups aimed to inform participants about the current guidelines (HP → IND). The focus groups also brought representatives of community organisations together to share information and discuss 'whole-of-community' nutrition strategies that would ultimately improve individual food choices (HP → [ORG-ORG] → IND). Thus there were two strategies employed in a single setting (the community);

Cummeragunja Women's Wellbeing Group: a weekly self-directed health-focused meeting for women at Cummeragunja facilitated RFNC staff, targeted the health and wellbeing of the women (HP → IND) and depended on the interaction between participants to share information and ideas (HP → [IND-IND]). In addition, this activity required sharing of resources between the two organisations (HP → [ORG-ORG] → IND). The setting for this activity was the organisation; and lastly

10-week body challenge: a workplace-based activity at Rumbalara Aboriginal Co-operative where a group of employees aimed to achieve 10,000 steps per day for 10 weeks facilitated by RFNC staff. The targets for this activity were three-fold: the workplace itself (eg. provision of pedometers and training to staff) (HP → ORG → IND), the outreach partnership between the sporting club and the workplace (HP → [ORG-ORG] → IND), and the staff themselves (HP → IND). Thus in total there were three strategies in a single setting.

As a whole, the entire program used five different strategies across two settings (organisation and community), receiving a score of 3 of a possible 4 (see Table 1). This shows a high level of consistency with the ecological approach [33,37]

## Discussion

To the best of our knowledge, this is the first attempt to extend the use of either store-turnover or ecological analysis to a semi-urban Australian Aboriginal community context. Overall, the Aboriginal health promotion program described here achieved a good degree of fit with the ecological model, suggesting that this community's model of health promotion has some congruence with ecological principles: it intervened at several levels, had multiple targets and used a number of varied strategies to address nutrition, physical activity and their determinants. To improve the ecological 'score' of the program, the number of settings in which health promotion activities take place would need to be increased to include 'society' and 'supranational' settings: in the former instance, this would address the Aboriginal community's relationship with mainstream society, identified as an important determinant of Aboriginal health [1]. However, we note that the current evaluation does not pick up on all the contextual factors within which the activities were developed. For example it does not consider Aboriginal Community-Controlled Organisations' (ACCOs) attempts to work with mainstream society to promote reconciliation and social inclusion. It would be useful to apply this analysis to the broader range of programs that are being conducted within the ACCOs.

The store turnover method showed that efforts by RFNC to model healthy eating behaviours led to changes in the food supply generally in line with recommendations [23]. Specifically, the variety of food was increased, as was the volume of fruit and vegetables, bread and lean meat. Total fat and sugars decreased in 2006 compared with 2005, and contributed similar proportions of energy to that reported in the Australian National Nutrition Survey: 17% protein, 35% total fat, 14% saturated fat, 48% total carbohydrate, 22% sugars [38]. Folate, vitamin C and fibre increased. Fat-soluble vitamins (A and E) were lower than ideal but we note these have only a modest association with chronic disease risk. Post-intervention, the food supply did not meet all of the characteristics of an optimal diet for chronic disease prevention according to the NHMRC nutrient reference values. However, given that these reference values are based on the 90^th ^percentile of intake for the Australian population rather than on observed relationships with chronic conditions, they provide rather a harsh basis for comparison [36]. Furthermore, the positive changes in food supply are significant in the context of a sports club, which have traditionally had poor quality food supplies [39,40], and because the RFNC is accessed by a large number of community members of varying age, gender and socio-economic status who gather to participate in community activities (including but not limited to sport). The RFNC is therefore a vehicle for changing community norms relating to nutrition.

We acknowledge that a focus on nutrients has limitations - people eat foods not nutrients - but this was necessary for comparison with national guidelines. An examination of the social and cultural meanings of the foods supplied, while of relevance, was beyond the scope of the present study. However, ethnographic research in an urban Aboriginal setting has emphasised the ways in which food, eating and exercise can act to connect or disconnect Aboriginal people from family and community [7]. Importantly, the current changes were made in a manner and setting that supported social connectedness, not disrupting this cultural imperative as individual dietary plans can.

This was a pilot study limited by its small scale and relatively short time-frame. The application of Richard et al.'s [33] analytical procedure to one program comprising a number of activities departs somewhat from the studies assessing multiple programs for which the method has typically been applied [37,41]. However, we would argue that this is a legitimate and useful application of the method as it allows practitioners to use the information to improve their own practice more directly by incorporating the findings into the reflection and planning phases of participatory research cycles, identifying leverage points for change and monitoring change in ecological 'score' over time. We note that since Richard et al. [33] developed their analytical procedure, subsequent work has sought to simplify the procedure by discarding the category of 'settings' and focusing on levels and agents of change [41]. Further work is required to better adapt the method for use in the community context and to incorporate indicators of health and its determinants specific to Aboriginal communities.

## Conclusion

The two evaluation methods described here made use of data accessed with minimal additional work demanded of program facilitators. While not disputing the importance of individual-level data, this evaluation was useful for assessing the impact of the program on the nutrition environment and for understanding the ecological nature of program activities. This in turn is useful information for planning subsequent activities. An advantage of the ecological approach is that it better allows evaluation of a holistic model of health service delivery. Although originally based on western social systems, the methodology has the potential to be adapted to local models of health and wellbeing which include community and social factors. The research outcomes therefore have relevance beyond primary healthcare to other sectors.

## Competing interests

The authors declare that they have no competing interests.

## Authors' contributions

RR contributed to the design of the study, analysed the 'ecological' data and drafted the manuscript; MC (2^nd ^author) conducted the store-turnover analysis and drafted the store-turnover methods and results sections; JD and BF designed and implemented interventions, contributed to the overall design of the study, collected and assisted with interpretation of data; MC (5^th ^author) contributed to the design and interpretation of the ecological analysis; GV and DM contributed to the design and implementation of interventions and assisted with data collection; and KR collaborated on all aspects of program design, data analysis and reporting. The Heart Health Project Steering Committee comprises senior members of the Aboriginal community who guided and made final decisions relating to all aspects of the project. All authors contributed to re-drafting and approved the manuscript.

## Pre-publication history

The pre-publication history for this paper can be accessed here:

http://www.biomedcentral.com/1471-2458/11/749/prepub
